# Effect of timing of surgical resection of primary hepatocellular carcinoma on survival outcomes in elderly patients and prediction of clinical models

**DOI:** 10.1186/s12876-021-01815-4

**Published:** 2021-05-21

**Authors:** Yongfei He, Tianyi Liang, Shutian Mo, Zijun Chen, Shuqi Zhao, Xin Zhou, Liping Yan, Xiangkun Wang, Hao Su, Guangzhi Zhu, Chuangye Han, Tao Peng

**Affiliations:** Department of Hepatobiliary Surgery, The First Affiliated Hospital of Guangxi Medical University, Nanning, 530021 Guangxi Province China

**Keywords:** Hepatocellular carcinoma, The elderly, Surgery, Nomogram model

## Abstract

**Background:**

The effect of time delay from diagnosis to surgery on the prognosis of elderly patients with liver cancer is not well known. We investigated the effect of surgical timing on the prognosis of elderly hepatocellular carcinoma patients undergoing surgical resection and constructed a Nomogram model to predict the overall survival of patients.

**Methods:**

A retrospective analysis was performed on elderly patients with primary liver cancer after hepatectomy from 2012 to 2018. The effect of surgical timing on the prognosis of elderly patients with liver cancer was analyzed using the cut-off times of 18 days, 30 days, and 60 days. Cox was used to analyze the independent influencing factors of overall survival in patients, and a prognostic model was constructed.

**Results:**

A total of 232 elderly hepatocellular carcinoma patients who underwent hepatectomy were enrolled in this study. The cut-off times of 18, 30, and 60 days were used. The duration of surgery had no significant effect on overall survival. Body Mass Index, Child-Pugh classification, Tumor size Max, and Length of stay were independent influencing factors for overall survival in the elderly Liver cancer patients after surgery. These factors combined with Liver cirrhosis and Venous tumor emboli were incorporated into a Nomogram. The nomogram was validated using the clinical data of the study patients, and exhibited better prediction for 1-year, 3-year, and 5-year overall survival.

**Conclusions:**

We demonstrated that the operative time has no significant effect on delayed operation in the elderly patients with hepatocellular carcinoma, and a moderate delay may benefit some patients. The constructed Nomogram model is a good predictor of overall survival in elderly patients with hepatectomy.

## Introduction

Primary hepatocellular carcinoma (HCC) is a common malignancy of the digestive tract worldwide and the second leading cause of cancer-related deaths [1]. Data shows that the new cases of liver cancer in China account for 45% of the new cases in the world annually, among which the incidence of elderly patients show an increasing trend year by year [2]. Presently, early intervention therapy is recommended for primary liver cancer to prevent further progression of the disease and achieve a better prognosis. However, several retrospective studies have demonstrated that 30% of the patients with liver cancer have experienced delayed clinical treatment [3, 4]. In clinical practice, several factors such as patients underlying diseases, psychological factors, and waiting for hospital beds, vary the duration of time to surgery (TTS) from the date of diagnosis of HCC to the date of operation.

Currently, only a few scholars have studied and analyzed the impact of delayed surgical treatment on the prognosis of patients with HCC, of which only one study employed surgery as the only intervention factor [4,6]. Most studies have shown that delaying the time of intervention treatment leads to poor prognosis of patients [3]. However, recent studies have shown that a moderate delay in the treatment of liver cancer patients does not affect the prognosis of patients [5], which is similar to the results obtained in lung cancer, thyroid cancer, and colorectal cancer [7].

There are no guidelines that explicitly recommend the timing of surgery for patients with HCC after diagnosis. The number of elderly HCC patients expected to require treatment is likely to continue to rise, given the rising life expectancy and aging populations around the world. There are different clinicopathologic features between the elderly patients and young patients with HCC, particularly for elderly patients with HCC the treatment delay is caused by the particularity of physiology and psychology of the body. Therefore, determining whether TTS affects the prognosis of elderly HCC patients will be significant in aiding the clinical decision-making and the rational allocation of medical resources. Hence, the present study investigated the effects of surgical duration on overall survival (OS) in the elderly (60 years) patients, and established a clinical model for the validation and prediction.

## Methods

### Object of study

The data of elderly patients with primary HCC who underwent hepatectomy in the First Affiliated Hospital of Guangxi Medical University from 2012 to 2018 was collected retrospectively. All the patients signed an informed consent approved by the hospital ethics committee, in line with the provisions of medical ethics. The diagnosis of primary HCC referred to the *Guidelines for diagnosis and treatment of primary liver cancer in China (2019 edition)* [8]. The inclusion criteria for patients were: (1) age60 years; (2) postoperative pathological diagnosis was confirmed as HCC; (3) preoperative assessment was that the HCC could be resected surgically. The exclusion criteria included: (1) secondary HCC; (2) early intervention for HCC (e.g., transcatheter arterial embolization, radiofrequency ablation, and portal venous embolization); (3) rupture of HCC and hemorrhage; and (4) postoperative follow-up loss.

## Data collection

Data from 232 cases were eventually obtained based on the inclusion and exclusion criteria. The patient data were collected through the hospital information management system. Collected data included personal information (gender, age, etc.), the background causes (presence of hepatitis b, cirrhosis, and alcohol, etc.), imaging (venous tumor emboli, size, and location of the tumor, single or multiple, etc.), intraoperative situation (operation method, operating time, tumor resection, amount of blood loss, and blood transfusion, etc.), pathology (degree of differentiation, liver cirrhosis, and microvascular invasion, etc.), the postoperative complications experienced and the length of hospital stay, etc. Postoperative complications were classified according to ClavienDindo [9] and recorded during hospitalization for the same operation or within 30 days after discharge. OS was defined as the time from surgery to death or the date of the last follow-up. The date of primary liver cancer was considered as the date of diagnosis for the initial imaging examination. Resectability was assessed based on the results of the imaging examination. TTS was defined as the number of days between the date of diagnosis and the day of surgery.

## Risk characteristics and prognosis

To assess the factors affecting OS of the patients, Cox regression analysis was conducted. After determining the coefficients of the influencing factors, a risk score prediction model was constructed based on the median risk score and HCC was divided into the high-risk and low-risk groups. Receiver operating characteristic (ROC) curves were used to detect the predictive efficiency of the survival model. Major factors influencing the OS of HCC patients were screened out, and the model was used for self-verification.

### Statistical analysis

Descriptive statistics were used to summarize the characteristics of the clinical data of the patients, which were expressed as the median [quartile range (IQR)] and frequency (%) of the continuous and categorical variables, respectively. Cox regression analysis was used to evaluate the relationship between the clinical data and OS of the elderly patients with liver cancer, and independent risk factors for OS were obtained. Then, TTS were grouped according to time points 18, 30, and 60 days, and the survival difference of different groups was compared by the KaplanMeier method, respectively. According to the selected independent risk factors, the subgroup analysis and the combination of Liver cirrhosis and Venous tumor emboli were used to establish the line chart prediction model. The data of the patients were used to verify the ROC curve (P<0.05 was considered to be statistically significant). Statistical analysis was performed using SPSS 23.0 and R software.

## Result

A total of 232 cases were included in this study, of which males accounted for the majority (75%) and the median age was 62 (64-67.8) years. In terms of diet and bad habits, more patients were associated with smoking (29.7%) and drinking history (28.4%), and 9.5% were associated with liver paragonimiasis. Regarding the metabolic diseases, 14.7% of patients had diabetes. Moreover, in terms of liver disease background, 69.4% of the patients were complicated with viral hepatitis B and 3.9% with viral hepatitis C, but 95.7% of the patients were classified as Child-Pugh Class A. Laparoscopic hepatectomy was performed in 23.3% of the patients, radical resection was performed in 59.5%, and intraoperative chemotherapy was used in 31.5%. The pathological results showed that 9.5% of the patients had cirrhosis, 27.6% had microvascular invasion, 12.1% had venous thromboembolism, and 88.4% were highly differentiated. Severe complications occurred in 2.6% of the patients, with a median hospital stay of 18 (1422) days. The median OS of patients was 37 (23.457.6) months (Table1).

**Table 1:** Characteristics of elderly patients with hepatocellular carcinoma

Variables	Overall (N=232)
Age*	62 (6467.8)
*Sex*	
Male	174 (75%)
Female	58 (25%)
BMI*	22.7 (20.624.8)
*Smoking*	
Yes	69 (27.9%)
No	163 (70.3%)
*Alcohol*	
Yes	66 (28.4%)
No	166 (71.6%)
*Diabetes*	
Yes	34 (14.7%)
No	198 (85.3%)
*BCLC stage*	
0A stage	190 (81.9%)
BC stage	42 (18.1%)
*Child-Pugh*	
Grade A	222 (95.7%)
Grade B	10 (4.3%)
*Liver cirrhosis*	
Yes	108 (46.6%)
No	124 (53.4%)
*Liver fluke*	
Yes	22 (9.5%)
No	210 (90.5%)
*Viral hepatitis B*	
Yes	161 (69.4%)
No	71 (30.6%)
*Viral hepatitis C*	
Yes	9 (3.9%)
No	223 (96.1%)
*AFP, ng/mL*	
8	65 (28%)
>8	167 (72%)
*Tumor number*	
Single	202 (87.1%)
Multiple	30 (12.9%)
*Tumor size, cm*	
<5	132 (56.9%)
5	100 (43.1%)
*Venous tumor emboli*	
Yes	28 (12.1%)
No	204 (87.9%)
Time of operation*	240 (172296)
Bleeding*	350 (200600)
*Local chemotherapy*	
Yes	73 (31.5%)
No	159 (68.5%)
*Radical resection*	
Yes	139 (59.5%)
No	94 (40.5%)
*Laparoscopic hepatectomy*	
Yes	54 (23.3%)
No	178 (76.7%)
*Differentiated degree*	
High	14 (6%)
Middle	205 (88.4%)
Low	13 (5.6%)
*Microvascular invasion*	
Yes	64 (27.6%)
No	168 (72.4%)
Length of stay*	18 (1422)
*ClavienDindo*	
I and II	226 (97.4%)
III, IV and V	6 (2.6%)
Overall survival*	37 (23.457.6)
*Survival status*	
Alive	184 (79.3%)
Death	48 (20.7%)
*Recurrence*	
Yes	69 (29.7%)
No	163 (70.3%)
TTS*	18 (1429.8)

*Median (IQR).AFP indicates alpha-fetoprotein;BMI, body-mass index;IQR, interquartile range

**Fig. 1:**
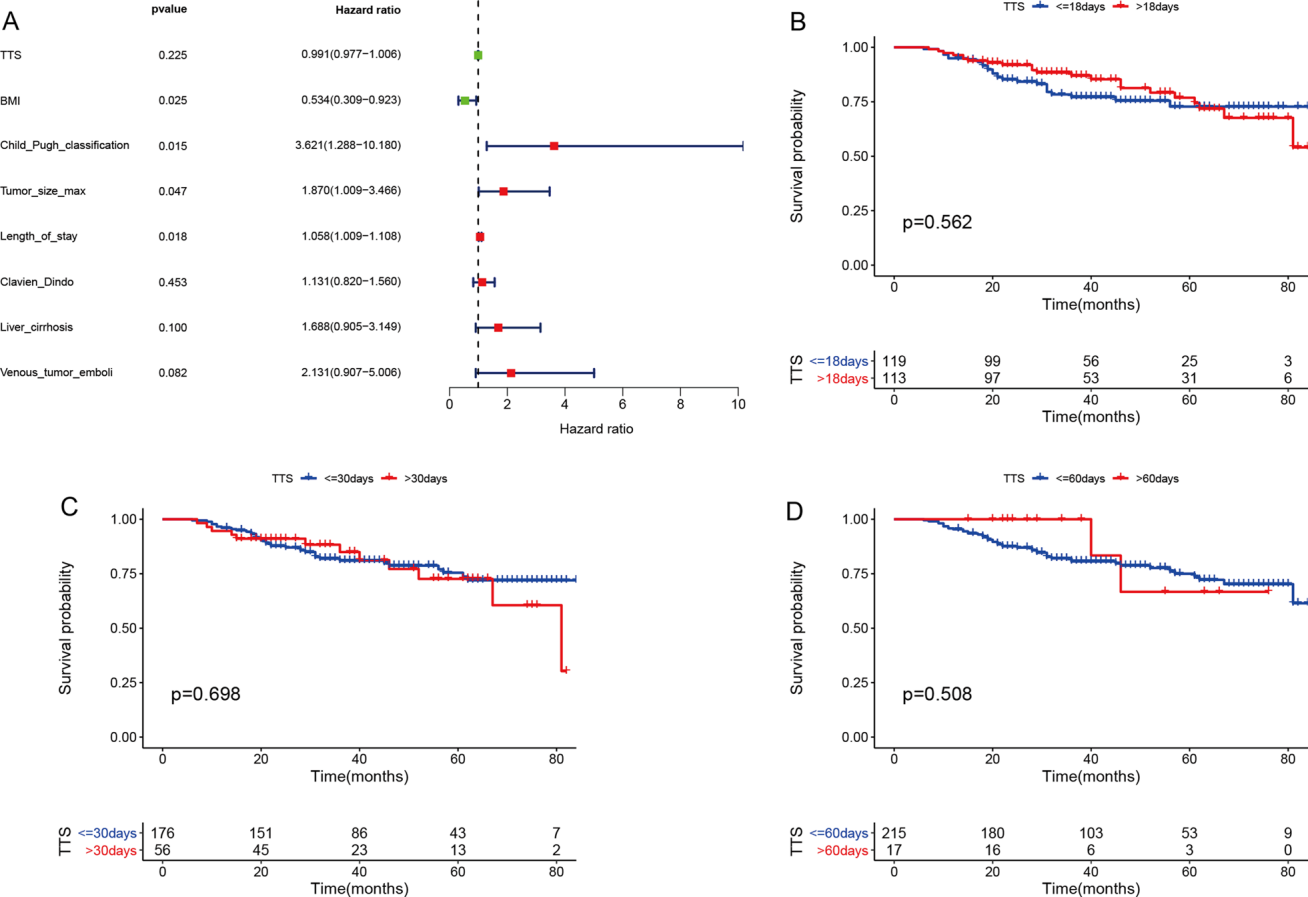
Multivariate Cox regression analysis affecting OS and KaplanMeier survival curves for different TTS. **a**Multivariate Cox regression analysis of postoperative OS in elderly patients with hepatocellular carcinoma. **b** TTS is the 18-day Kaplan-Meier survival curve. **c** TTS is the 30-day KaplanMeier survival curve. D, TTS is the 60 - day Kaplan-Meier survival curve

To determine the factors related to the OS of the patients, univariate analysis was performed. The results showed that Body Mass Index (BMI), Child-Pugh Classification, Tumor size Max, Clavien Dindo, and Length of stay were related to the prognosis of the patients, and TTS did not significantly affect the OS of the patients (Table2). Subsequently, TTS were included in Cox multivariate analysis, and the results further confirmed that TTS was independent of the OS of the patients and that BMI, Child-Pugh Classification, Tumor size Max, and Length of stay were independent risk factors for OS in elderly patients with liver cancer after surgery (Fig.1a). We divided TTS into three-time blocks of 18 days, 30 days, and 60 days to analyze the OS of the patients. The results showed that within 60 days of TTS, there was no significant difference in the survival curve of the OS with the increase of TTS (Fig.1bd).

**Table 2:** Univariate Cox regression analysis of OS in elderly patients with hepatocellular carcinoma after operation

Factors	HR (95% CI)	P value
BMI	0.425 (0.2440.741)	0.003
Child Pugh classification	3.112 (1.2297.878)	0.017
Tumor size max	2.02 (1.1373.591)	0.017
Clavien Dindo	1.341 (1.0271.751)	0.031
Length of stay	1.049 (1.0061.094)	0.024

**Fig. 2:**
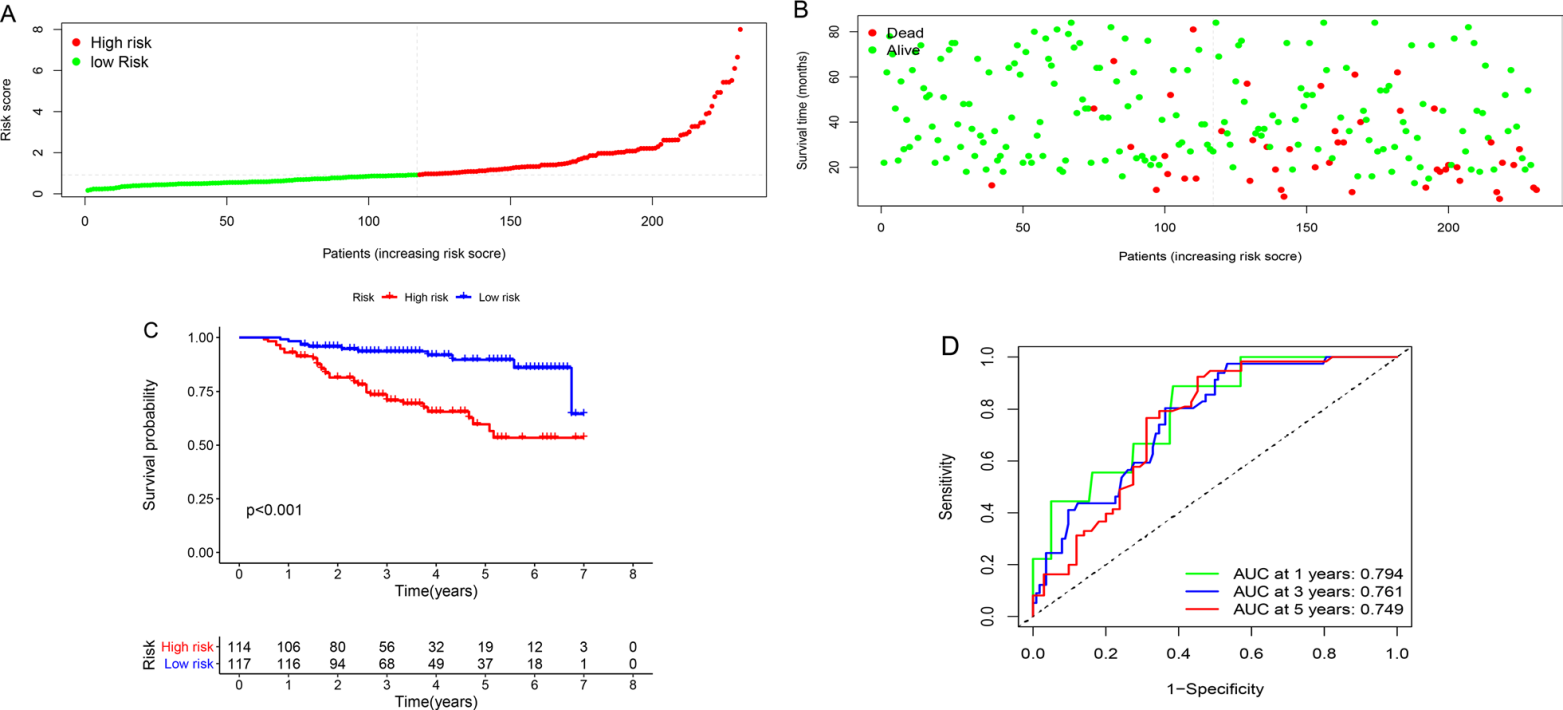
Prognostic risk score and predictive efficiency. **a** Distribution of risk scores. **b** Patient survival time and status. **c** Risk factor KaplanMeier survival curve. **d** The ROC curve shows the predictive efficiency of the risk score

Previous studies have shown that Liver cirrhosis and Venous tumor emboli are associated with the prognosis of patients with liver cancer [10], combined with the results of Cox multivariate analysis affecting OS. the risk score formula for predicting OS was calculated from the above six factors: Risk score = (Child Pugh classification*1.13) + (Tumor size Max *0.71) + (Length of stay*0.06) + (Liver observing group) + (BMI* 1.13) + (Venous Tumor emboli*0.57) (BMI*0.68). Depending on the median risk, patients were classified into the high-risk and low-risk groups, and the distribution of risk score and the survival time and status of the patient were observed as shown in Fig.2a, b. Survival analysis showed that patients in the high-risk group had a worse OS than those in the low-risk group (P<0.001) (Fig.2c). We then used the receiver operating characteristic curve (ROC) analysis to assess the prognostic value of these factors. The results showed that the area under ROC curve (AUC) of 1-year, 3-year, and 5-year OS was 0.794, 0.761, and 0.749 respectively (Fig.2d).

**Fig. 3:**
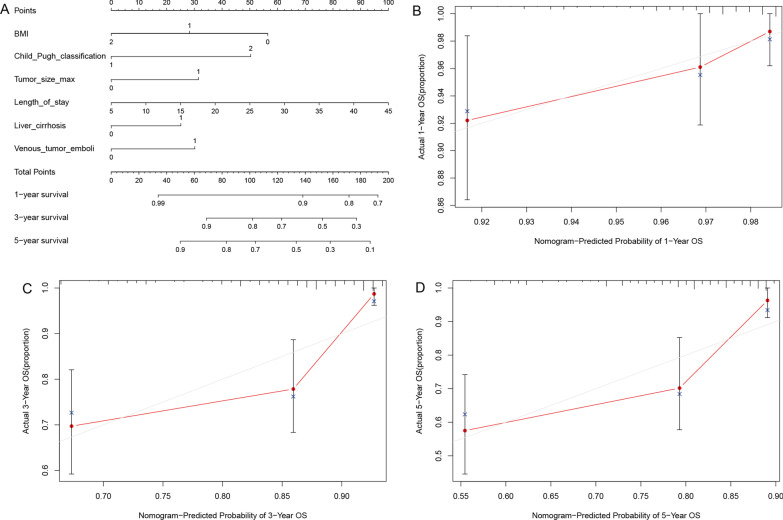
Nomogram prediction model and prediction OS curve. **a** Nomogram predicts a 1-year OS curve. **b** Nomogram predicts a 3-year OS curve. **c** Nomogram predicts a 5-year OS curve

**Fig. 4:**
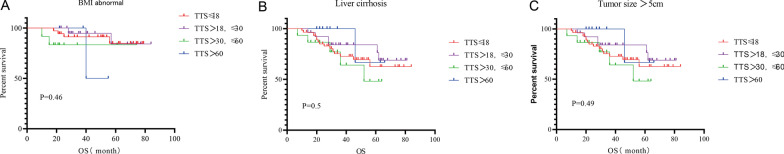
The relationship between single factor TTS and OS. **a** The relationship between different TTS and OS in abnormal BMI. **b** The relationship between different TTS and OS in patients with cirrhosis. **c** The relationship between different TTS and OS at tumor>5cm

Based on the above risk factors, we established a nomogram prediction model (Fig.3a). Subsequently, the model was validated using the clinical data of the study patients. The results showed that the optimal time between model prediction and actual observation was 1 year OS after surgery, followed by 3 years OS, and 5 years OS (Fig.3bd). Subsequently, we performed subgroup analysis of ABNORMAL BMI, cirrhosis, and Tumor size, and the results showed that there was no significant difference in OS among patients with different TTS (Fig.4).

## Discussion

In the secondary prevention of tumors and various guidelines, the treatment attitude towards tumors has always advocated early detection, early prevention, and early treatment [8, 11, 12], and most types of literature have also pointed out that delayed treatment is not conducive to the prognosis of patients. In a large retrospective study involving 26,038 patients with liver cancer, according to the results in the diagnosis of more than 181 and 61180 days after the treatment of patients with a 1.68 -fold increased risk of death (95% HR 1.501.88), a 1.39-fold increased risk of death (95% HR 1.311.17), for early HCC patients, from diagnosis to treatment for a longer time interval would result in a lower survival rate [13]. Besides, several studies have shown that delayed treatment increases the risk of residual tumor and death, and delayed treatment of HCC is associated with shortened OS, and significantly delayed treatment leads to worse survival rates, especially in patients with delayed treatment of more than 3 months [3, 14,17]. This is contrary to the results obtained in this study. Contrary to the results of our study, it may be related to the different time of the definition of delayed treatment and the treatment measures are taken, because the time point of delayed treatment defined by our study is shorter than that of the above study. closer to the real clinical scene of the center.At the same time, the present study included only surgically resected patients, and the treatment measures in the other studies included radiofrequency ablation, transarterial chemoembolization, percutaneous ethanol injection, and liver transplantation.

Several recent studies have shown that delayed treatment does not increase the risk of early death in HCC patients, but rather may reduce the risk of death, and does not affect the prognosis of HCC patients with early liver cancer [4, 5]. A cohort study of 12,102 patients with HCC conducted by Croome et al. showed that patients with TTS more than 60 days after hepatectomy had a lower risk of death and a higher 5-year survival rate [15], The authors speculate that patients with severe disease and a higher risk of late death may undergo early surgery, while patients with lower invasiveness will receive surgery later. And receive a more comprehensive preoperative evaluation.The Tousif Kabir et al. study was the most similar to this study regarding the design [18], in which they used time cut-off points of 30, 60, and 90 days in the analysis, and compared OS between each group and reported no significant differences. We also only reviewed the relationship between the different TTS and OS according to the different time cut-off points, but the selection of the time cut-off point was different. The median waiting time for liver cancer patients in our center is 18 days, while the waiting time for liver cancer patients in western countries is about 3 months, which is related to different medical systems [3, 15, 19, 20]. The TTS time we selected for 18 days, 30 days, and extension to 60 days were similar in OS and did not yield statistical significance.TTS had no significant effect on OS and suggested that moderate extension of TTS appeared to be beneficial. All the patients we included in the analysis were elderly patients, with better pertinence and population representation.

A subgroup analysis of the factors associated with tumor prognosis was conducted. Results showed that increase in TTS did not change the OS significantly in patients with abnormal BMI. Studies have showed that nutrition treatment for patients before liver tumor resection is helpful in improving their tolerance to surgery and reducing postoperative hospital stay [21, 22]. Therefore, it is important to correctly evaluate the liver function when performing hepatocellular carcinoma resection, especially for elderly patients with liver cirrhosis, where the structural metabolism and function of liver cells decline, and abrupt hepatectomy is likely to lead to acute liver insufficiency or even liver failure [23]. Our analysis of patients with cirrhosis showed that a modest increase in TTS did not affect the OS of the patients. Preoperative treatment to improve the liver function of such patients may reduce the occurrence of such complications [24]. Many studies have indicated that the tumor size is correlated with the prognosis of patients ^[14,2528]^, and this phenomenon has also been observed in our study. Our further analysis showed that patients with a tumor diameter of >5cm did not show worse OS with the increase of TTS. This is very helpful for clinicians because, for patients with large left and right liver tumors, one-stage radical hepatectomy or even tumor resection often cannot be performed because of insufficient residual liver volume. it is necessary to compensate for the reserved liver hyperplasia by means of two-step hepatectomy combined with hepatic parenchyma separation and portal vein ligation and portal vein embolization [29, 30]. Our study provides a theoretical basis for patients who need two-step liver resection and improves the resection rate of HCC.

In this study, we established a post-operative nomogram based on four independent factors including cirrhosis and venous thrombi that affect OS using a Cox multivariate analysis. Using the model, each elderly HCC patient was assigned a unique nomogram score to predict postoperative survival. The lower the score, the better the OS, and surgical treatment was recommended for such patients. Because these variables are readily available postoperatively, they provide an easy to use tool for individualized and comprehensive prognostic assessment. Our results suggest that elderly HCC patients with higher nomogram scores have poorer postoperative survival outcomes. The patients should be followed up to assess tumor recurrence in a timely manner and initiate treatment, or to provide adjuvant therapy to improve the quality of life.

However, this study had limitations. Because this study was a single-center retrospective analysis and was susceptible to selection bias, some patients who lost the opportunity for surgery during tumor progression while waiting for treatment, or who did not choose surgical treatment for various reasons, were excluded. In addition, in terms of the construction of the clinical model, we selected several factors affecting OS and a small number of clinical indicators, and used our own data for verification, without large-scale external data.

## Conclusions

We specifically studied the effect of delayed surgery on the prognosis of elderly hepatocellular carcinoma patients. Appropriate delayed surgery may benefit some patients, fully improve the organ status of the patient, and optimize the concurrent diseases, to reduce the surgical risk and improve the prognosis. The model can be used to predict the prognosis of patients.

## Data Availability

The data and materials used to support the findings of this study are available from the corresponding author upon request.

## Abbreviations

HCC: Hepatocellular carcinoma
TTS: Time to surgery
OS: Overall survival
BMI: Body Mass Index
ROC: Receiver operating characteristic
